# Endoplasmic Reticulum Stress Induced by Turbulence of Mitochondrial Fusion and Fission Was Involved in Isoproterenol-Induced H9c2 Cell Injury

**DOI:** 10.3390/ijms27031390

**Published:** 2026-01-30

**Authors:** Shengnan Zhang, Liqin Chen, Fuquan Jia, Shuguang Zhang, Huimin Zhang, Weibo Shi, Bin Cong

**Affiliations:** 1 College of Basic Medical Science, Inner Mongolia Medical University, Hohhot 010059, China; 2 Hebei Key Laboratory of Forensic Medicine, Collaborative Innovation Center of Forensic Medical Molecular Identification, Department of Forensic Medicine, Hebei Medical University, Shijiazhuang 050017, China

**Keywords:** ISO, mitochondria fusion, mitochondria fission, endoplasmic reticulum stress, cardiomyocyte injury

## Abstract

Alterations in mitochondrial fusion and fission dynamics are critical determinants of cellular fate. However, how stress-induced mitochondrial fusion and fission affect the physiological and pathological processes in cardiomyocytes remains poorly understood. Based on an established in vitro model of stress-induced cardiomyocyte injury using isoproterenol-treated H9c2 cells, this study aimed to investigate whether the dysregulation of mitochondrial dynamics—specifically, an imbalance between fusion and fission—activates the IRE1α-ASK1-JNK endoplasmic reticulum stress signaling pathway, thereby contributing to cardiomyocyte damage. Under this experimental paradigm, cell viability was evaluated using the CCK-8 assay. Concurrently, immunofluorescence staining was employed to assess reactive oxygen species accumulation, the expression of key mitochondrial fusion/fission proteins, and components of the ER stress pathway (IRE1α, ASK1, and JNK). Results demonstrated that isoproterenol treatment elevated intracellular ROS levels and induced significant changes in both mitochondrial dynamics-related proteins and the IRE1α-ASK1-JNK signaling axis. In contrast, administration of the mitochondrial fission inhibitor Mdivi-1 attenuated ROS accumulation, restored the expression of the affected proteins toward normal levels, and alleviated cardiomyocyte injury. Collectively, these findings indicate that the disruption of mitochondrial fusion/fission dynamics triggers endoplasmic reticulum stress via the IRE1α-ASK1-JNK cascade, which participates in the pathological progression of cardiomyocyte injury.

## 1. Introduction

Physiological stress functions as a double-edged sword: moderate levels are essential for adaptive physiological processes, whereas excessive or chronic exposure may trigger maladaptive reactions that disrupt systemic homeostasis. Substantial and growing evidence confirms the role of stress in the pathogenesis of a spectrum of disorders involving multiple organ systems, notably the nervous, cardiovascular, and immune systems [1,2,3]. These effects are primarily mediated by the activation of the hypothalamic–pituitary–adrenal (HPA) axis and the sympathetic–adrenal–medullary (SAM) axis, which regulate the release of stress hormones and orchestrate a broad range of physiological adjustments, thereby modulating emotional states, behavioral patterns, energy metabolism, and immune function [4,5]. Importantly, sustained sympathetic overactivation—characterized by excessive catecholamine release—is widely acknowledged as a critical contributor to cardiac injury and the progression of cardiovascular diseases. However, the precise molecular mechanisms and cellular pathways through which stress directly damages cardiomyocytes remain incompletely understood.

Mitochondria, serving as the central organelles within cardiomyocytes, sustain the heart’s high energy demands, accounting for approximately one-third of the total cellular volume [6,7]. These highly dynamic structures not only synthesize ATP but also sense stress signals and undergo morphological remodeling to adapt to metabolic alterations. Mitochondrial functional homeostasis relies on a dynamic equilibrium between fusion and fission—a quality-control mechanism critical for maintaining normal physiological activity [8]. Through the modulation of their size, distribution, and network architecture, mitochondria continuously adjust their fusion/fission dynamics to fulfill fluctuating energy demands [9]. Under physiological conditions, mitochondrial fission is initiated primarily through the phosphorylation of Drp1 at Ser616 and its subsequent binding to Fis1, a process that facilitates the clearance of damaged mitochondria. Conversely, mitochondrial fusion is coordinated by the outer membrane proteins Mfn1 and Mfn2, along with the inner membrane protein Opa1, which collectively promote intramitochondrial content exchange and enhance ATP synthesis, thereby maintaining mitochondrial functional integrity [10]. Collectively, these dynamic morphological adaptations give rise to a highly plastic mitochondrial network [11,12]. Disruption of this network results in mitochondrial fragmentation, impaired oxidative phosphorylation, and the excessive accumulation of reactive oxygen species (ROS), thereby serving as a key mechanistic contributor to the development and progression of cardiovascular diseases [13].

Furthermore, through mitochondria-associated endoplasmic reticulum membranes (MAMs)—structures central to the coordinated regulation of calcium homeostasis, lipid metabolism, and energy transfer [14,15,16]—mitochondria establish close physical and functional connections with the endoplasmic reticulum (ER). This structural coupling establishes a tight functional interdependence between these two organelles. Under sustained stress, mitochondrial dysfunction—characterized by fragmentation, decreased membrane potential, and excessive ROS generation—could be directly transmitted via MAMs to compromise adjacent ER function. Specifically, ROS and dysregulated calcium signaling disrupt the redox balance and calcium homeostasis of the ER, leading to the accumulation of unfolded or misfolded proteins. This accumulation, in turn, triggers the activation of the unfolded protein response (UPR). Within the UPR, the IRE1α pathway is significantly activated as a key sensor branch—a response directly attributable to the MAM-mediated disruption of ER homeostasis resulting from mitochondrial dysfunction [17,18]. Activated IRE1α exerts dual functions: its endoribonuclease activity cleaves XBP1 mRNA to generate the transcriptionally active spliced form, sXBP1, while its kinase domain recruits the adaptor protein TRAF2, thereby initiating the ASK1-JNK signaling cascade [19,20]. Consequently, mitochondrial dysfunction, through MAM-mediated interorganellar communication, directly triggers an ER stress response characterized by sustained IRE1α activation. This leads to the recruitment of TRAF2/ASK1, activation of the JNK pathway, and ultimately results in cell death driven by JNK overexpression.

However, whether dysregulated mitochondrial fusion/fission dynamics participate in activating the endoplasmic reticulum stress pathway—particularly the IRE1α-ASK1-JNK axis—during ISO-induced cardiomyocyte injury remains unclear. To address this question, we established an in vitro injury model using H9c2 cardiomyocytes, aiming to elucidate the underlying molecular mechanisms.

## 2. Results

### 2.1. Morphological Characterization of Differentiated H9c2 Cardiomyocytes

**Figure 1:**
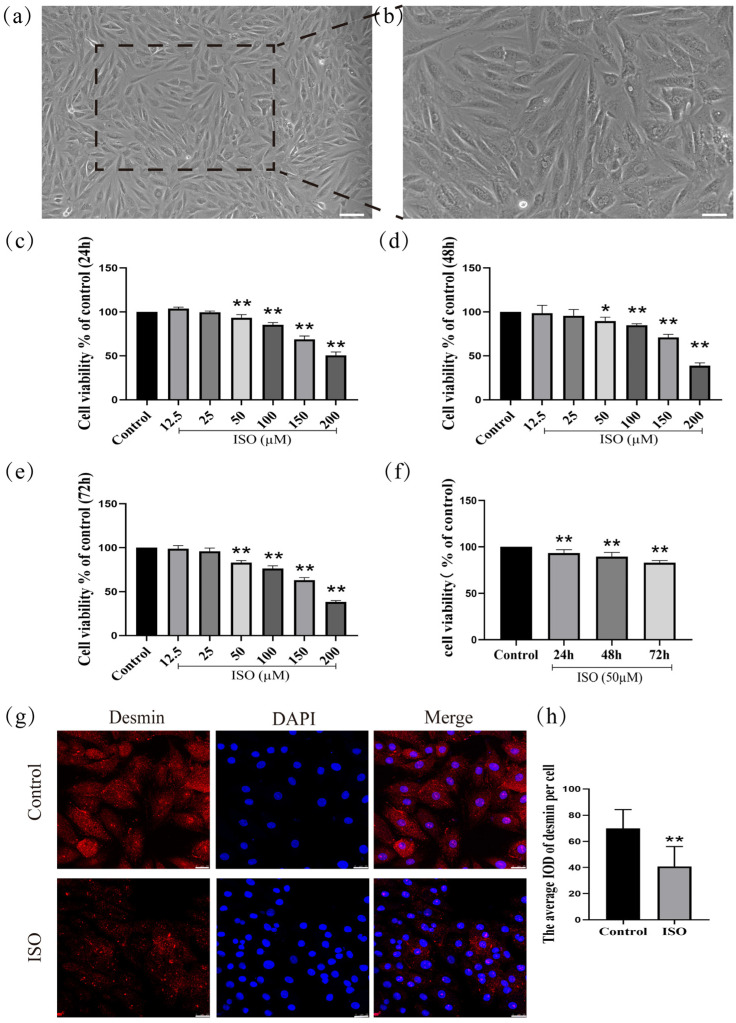
Establishment and validation of an ISO-induced injury model in H9c2 cardiomyocytes. (**a**) Scale bars = 200 μm or (**b**) scale bars = 50 μm. (**c**–**f**) The detection of cell viability. (**g**,**h**) The expression of desmin was detected in H9c2 cells by immunofluorescence. Scale bars = 25 μm. IOD = Integrated Optical Density. Data are presented as the mean ± SD (n = 6 independent biological experiments). * *p* < 0.05, ** *p* < 0.01 control vs. ISO-only group (one-way ANOVA with Tukey’s post hoc test).

H9c2 cardiomyocytes (derived from rat embryonic cardiac tissue) were cultured for 72 h in a medium containing 10% fetal bovine serum. After this period, the cells exhibited characteristic morphological features of differentiation, including an interconnected network, elongated spindle-shaped morphology, and translucent cytoplasm (Figure 1a,b).

### 2.2. Assessment of Viability in H9c2 Cardiomyocytes

To investigate the effects of ISO on H9c2 cells after treatment for 24 h, 48 h, and 72 h, ISO at different concentrations (12.5, 25, 50, 100, 150, and 200 µM) was initially administered to H9c2 cells. Then, cell viability was assessed by CCK-8 assay. As shown in (Figure 1c), one-way ANOVA results show that the viability of H9c2 cells was significantly decreased with the rise in ISO concentrations for 24 h (*F*(6, 41) = 289.15, *p* < 0.01). Post hoc comparisons shown that 12.5 µM (*p* > 0.01), 25 µM (*p* > 0.01), 50 µM (*p* < 0.01), 100 µM (*p* < 0.01), 150 µM (*p* < 0.01), and 200 µM (*p* < 0.01), compared with the control group. Compared with the control group, one-way ANOVA results show that the viability of H9c2 cells was significantly decreased with the rise in ISO concentrations for 48 h (*F*(6, 41) = 112.35, *p* < 0.01). Post hoc comparisons show that 12.5 µM (*p* > 0.01), 25 µM (*p* > 0.01), 50 µM (*p* < 0.01), 100 µM (*p* < 0.01), 150 µM (*p* < 0.01), and 200 µM (*p* < 0.01) (Figure 1d). Compared with the control group, one-way ANOVA results show that the viability of H9c2 cells was significantly decreased with the rise in ISO concentrations for 72 h (*F*(6, 41) = 400.17, *p* < 0.01). Post hoc comparisons show that 12.5 µM (*p* > 0.01), 25 µM (*p* > 0.01), 50 µM (*p* < 0.01), 100 µM (*p* < 0.01), 150 µM (*p* < 0.01), and 200 µM (*p* < 0.01) (Figure 1e). Compared with the control group (*F*(3, 23) = 32.40, *p* < 0.01), one-way ANOVA results show that the viability of H9c2 cells was significantly decreased for 24 h (*p* < 0.01), 48 h (*p* < 0.01), and 72 h (*p* < 0.01) after treatment with 50 µM (Figure 1f). Cell viability was assessed after treating H9c2 cardiomyocytes with ISO (12.5–200 µM) for 24 h, 48 h, or 72 h using the CCK-8 assay. The results demonstrated that ISO induced a significant reduction in cell viability in a concentration- and time-dependent manner.

### 2.3. Desmin Expression in H9c2 Cardiomyocytes

The expression of desmin, a cytoskeletal protein and specific biomarker of cardiomyocyte injury, was significantly downregulated following ISO treatment compared to the control group (*F*(1, 12) = 12.90, *p* < 0.01; Figure 1g,h), confirming ISO-induced cardiomyocyte injury.

### 2.4. Expression of Mitochondrial Fusion Proteins mfn1, mfn2, and opa1 in H9c2 Cardiomyocytes

**Figure 2:**
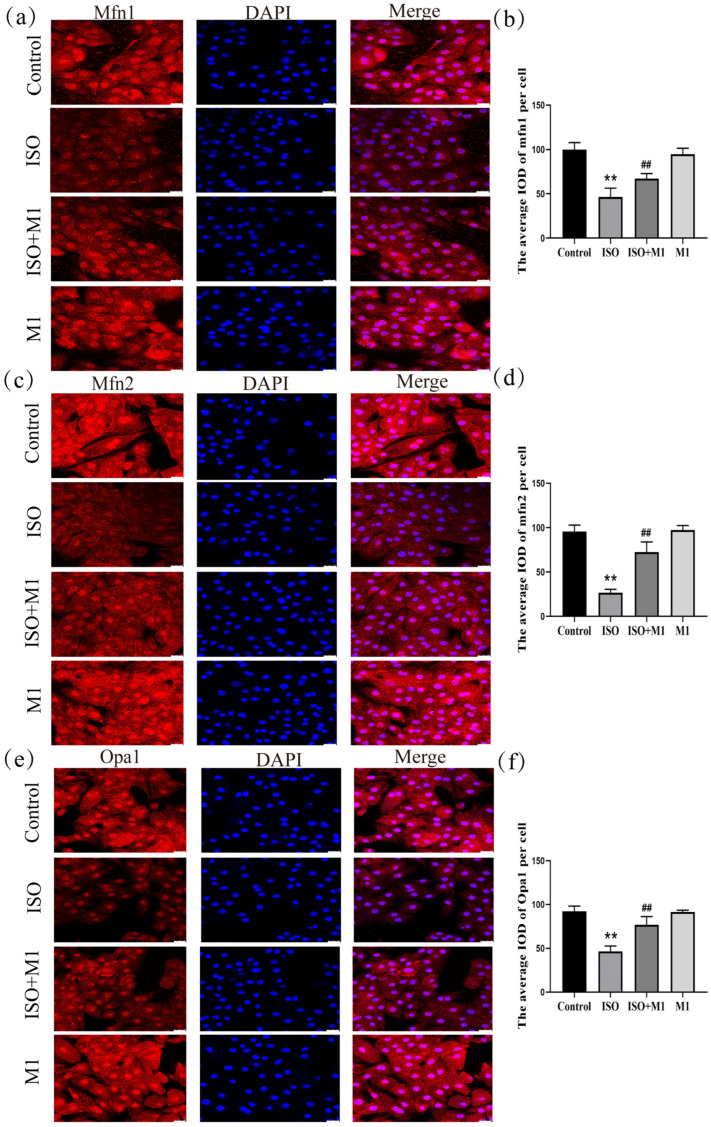
Expression of mitochondrial fusion proteins mfn1, mfn2, and opa1 in H9c2 cardiomyocytes. (**a**,**b**) The expression of mfn1 in H9c2 cells. Scale bars = 25 μm. (**c**,**d**) The expression of mfn2 in H9c2 cells. Scale bars = 25 μm. (**e**,**f**) The expression of opa1 in H9c2 cells. Scale bars = 25 μm. IOD = Integrated Optical Density. Data are presented as the mean ± SD (n = 6 independent biological experiments). ** *p* < 0.01 control vs. ISO-only group; ## *p* < 0.01 ISO vs. ISO + MI(Mdivi-1) group (two-way analysis of variance (ANOVA) with Tukey’s post hoc test).

The expression of key mitochondrial fusion proteins mfn1, mfn2 and opa1 was quantitatively assessed to evaluate mitochondrial dynamics under different treatment conditions. A two-way ANOVA of the mitochondria fusion protein revealed significant effects on the interaction among ISO combined with Mdivi-1 treatment fusion proteins [mfn1: (*F*(1, 24) = 16.91, *p* < 0.01); mfn2: (*F*(1, 24) = 52.93, *p* < 0.01); and opa1: (*F*(1, 24) = 33.82, *p* < 0.01)] in H9c2 cells, ISO treatment [mfn1: (*F*(1, 24) = 161.86, *p* < 0.01); mfn2: (*F*(1, 24) = 241.43, *p* < 0.01); and opa1: (*F*(1, 24) = 131.08, *p* < 0.01)] in H9c2 cells, and Mdivi-1 treatment [mfn1: (*F*(1, 24) = 6.04, *p* < 0.01); mfn2: (*F*(1, 24) = 61.16, *p* < 0.01); and opa1: (*F*(1, 24) = 31.09, *p* < 0.01)]. After ISO treatment, post hoc tests showed decreased mitochondria fusion protein (mfn1 (*p* < 0.01), mfn2 (*p* < 0.01), and opa1 (*p* < 0.01)). In contrast, ISO and Mdivi-1 treatment significantly increased the expression of mitochondria fusion protein [mfn1 (*p* < 0.01), mfn2 (*p* < 0.01), and opa1 (*p* < 0.01) (Figure 2a–f)]. A two-way ANOVA indicated that ISO treatment alone significantly suppressed the expression of all three fusion proteins. In contrast, pretreatment with the mitochondrial fission inhibitor Mdivi-1 prior to ISO exposure substantially counteracted this suppression and significantly elevated their expression levels. These results demonstrated that the ISO-induced impairment of mitochondrial fusion was effectively rescued by the pharmacological inhibition of fission, suggesting that excessive fission activity constitutes a primary mechanism through which ISO disrupts mitochondrial dynamics in H9c2 cardiomyocytes.

### 2.5. Expression of Mitochondrial Fission-Related Proteins Drp1^ser616^, Drp1, and Fis1 in H9c2 Cardiomyocytes

**Figure 3:**
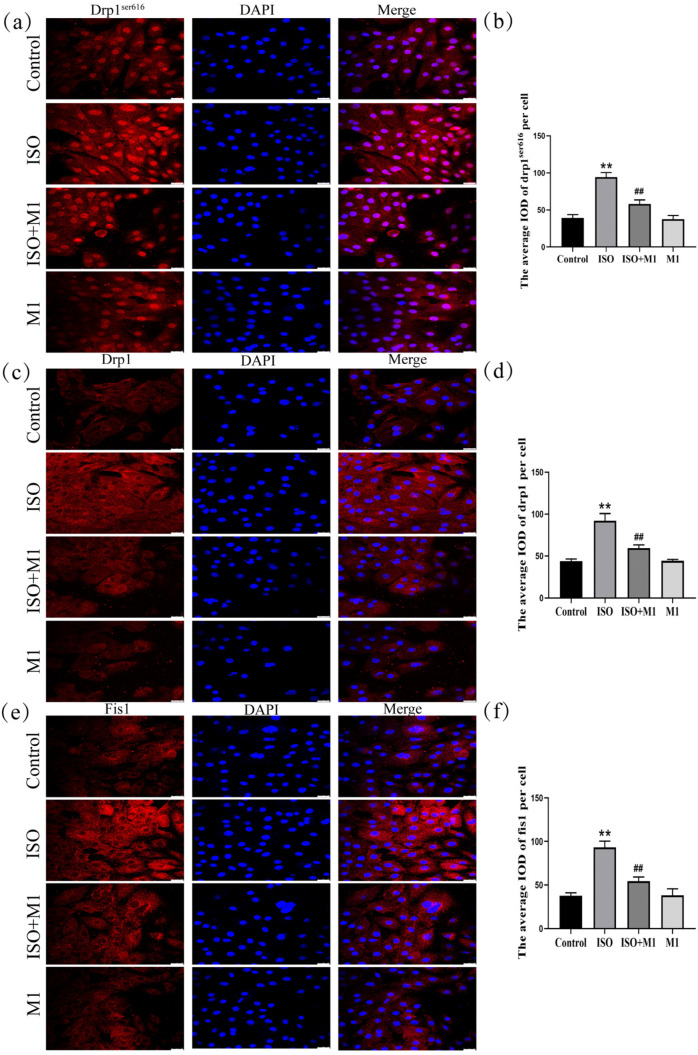
Expression of mitochondrial fission-related proteins Drp1^ser616^, Drp1, and Fis1 in H9c2 cardiomyocytes. (**a**,**b**) The expression of Drp1^ser616^ in H9c2 cells. Scale bars = 25 μm. (**c**,**d**) The expression of Drp1 in H9c2 cells. Scale bars = 25 μm. (**e**,**f**) The expression of fis1 in H9c2 cells. IOD = Integrated Optical Density. Scale bars = 25 μm. Data are presented as the mean ± SD (n = 6 independent biological experiments). ** *p* < 0.01 control vs. ISO group; ## *p* < 0.01 ISO vs. ISO + MI(Mdivi-1) group (two-way analysis of variance (ANOVA) with Tukey’s post hoc test).

To determine whether ISO drives the mitochondrial dynamic imbalance by regulating the expression of fission proteins, we systematically analyzed the expression levels of Drp1 and Fis1 following treatment with the mitochondrial fission inhibitor Mdivi-1. As shown in (Figure 3a–f), a two-way ANOVA of mitochondria fission protein revealed significant effects of ISO and Mdivi-1 treatment, ISO treatment, and Mdivi-1 treatment alone on the fission protein [drp1^ser616^: (*F*(1, 24) = 67.22, *p* < 0.01), (*F*(1, 24) = 320.21, *p* < 0.01), and (*F*(1, 24) = 79.73, *p* < 0.01); drp1: (*F*(1, 24) = 68.01, *p* < 0.01), (*F*(1, 24) = 251.93, *p* < 0.01), and (*F*(1, 24) = 64.43, *p* < 0.01); and fis1: (*F*(1, 24) = 67.11, *p* < 0.01), (*F*(1, 24) = 224.41, *p* < 0.01), and (*F*(1, 24) = 64.13, *p* < 0.01)]. After ISO treatment, post hoc tests showed increased fission protein (drp1^ser616^, drp1, fis1, and *p* < 0.01). In contrast, ISO and Mdivi-1 treatment significantly decreased fission protein (drp1^ser616^, drp1, fis1, *p* < 0.01). Post hoc analysis indicated that ISO treatment alone significantly increased the expression of all fission proteins examined (drp1^ser616^, total Drp1, and Fis1: all *p* < 0.01). In contrast, co-treatment with ISO and Mdivi-1 significantly reduced their expression (all *p* < 0.01). Consistent with the disruptive effect of ISO on mitochondrial dynamics, two-way ANOVA confirmed that ISO treatment markedly upregulated the expression of key fission proteins—phosphorylated Drp1 at Ser616 (drp1^ser616^), total Drp1, and Fis1-in H9c2 cardiomyocytes (all *p* < 0.01). However, this ISO-induced pro-fission effect was effectively reversed by pretreatment with the specific mitochondrial fission inhibitor Mdivi-1. Subsequent post hoc analysis further demonstrated that, compared to ISO treatment alone, co-treatment with ISO and Mdivi-1 significantly lowered the expression levels of all fission proteins measured (all *p* < 0.01). These results indicated that ISO induced an imbalance in the mitochondrial fusion/fission dynamics and that this disturbance could be alleviated by the inhibition of mitochondrial fission.

### 2.6. Expression of Tomm20, Cyto-c, and ROS in H9c2 Cardiomyocytes

**Figure 4:**
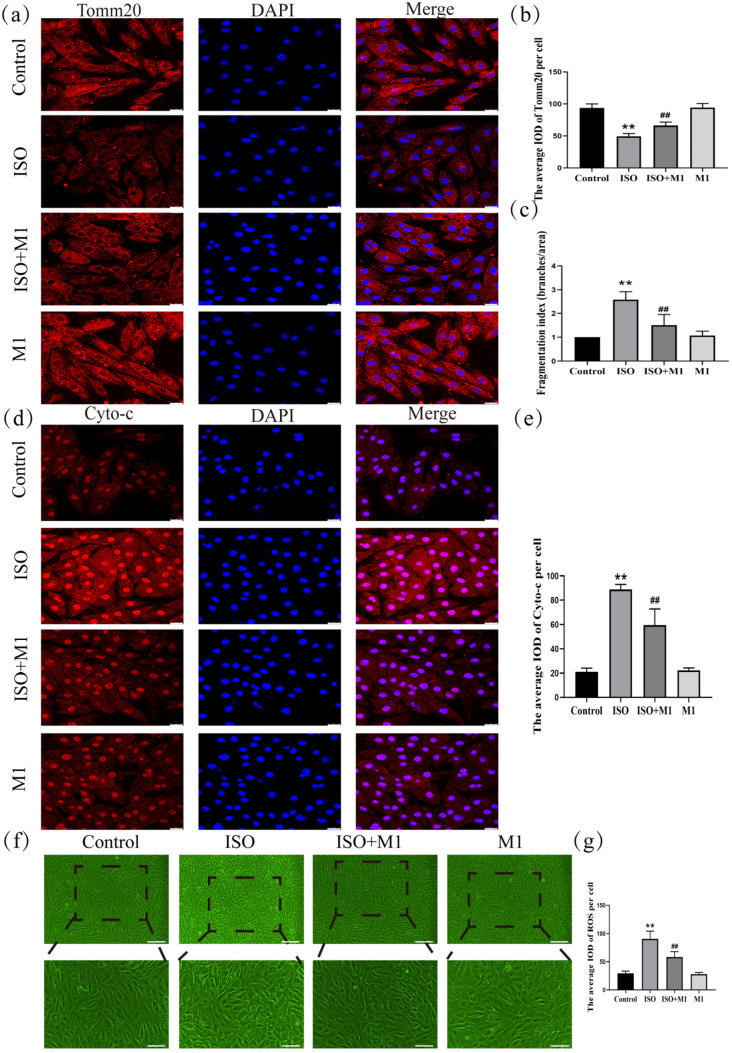
Expression of Tomm20, Cyto-c, and ROS in H9c2 cardiomyocytes. (**a**,**b**) The expression of Tomm20 in H9c2 cells. Scale bars = 25 μm. (**c**) Fragmentation Index (Branches/Area) = number of Branches/Network Area. Scale bars = 25 μm. (**d**,**e**) The expression of Cyto-c in H9c2 cells. Scale bars = 25 μm. (**f**,**g**) The expression of ROS in H9c2 cells. Scale bars = 25 μm. IOD = Integrated Optical Density. Data are presented as the mean ± SD (n = 6 independent biological experiments). ** *p* < 0.01 control vs. ISO group; ## *p* < 0.01 ISO vs. ISO + MI(Mdivi-1) group (two-way analysis of variance (ANOVA) with Tukey’s post hoc test).

To precisely assess mitochondrial morphology, this study employed translocase of the outer mitochondrial membrane 20 (Tomm20) as the marker protein. Thus, by performing a skeleton analysis on the Tomm20 signal, we quantified the mitochondrial network’s Fragmentation Index, which served as an objective metric to evaluate mitochondrial structural remodeling [21,22]. And a two-way ANOVA of the Fragmentation Index revealed significant effects on the interaction among ISO combined with Mdivi-1 treatment [Fragmentation Index: (*F*(1, 24) = 15.034, *p* < 0.01)] in H9c2 cells, ISO treatment [Fragmentation Index: (*F*(1, 24) = 47.121, *p* < 0.01)] in H9c2 cells, and Mdivi-1 treatment [Fragmentation Index: (*F*(1, 24) = 11.52, *p* < 0.01)]. After ISO treatment, post hoc tests showed an increased Fragmentation Index (*p* < 0.01). In contrast, ISO and Mdivi-1 treatment significantly decreased the Fragmentation Index (*p* < 0.01) (Figure 4c). These morphometric analyses revealed that stress treatment induced excessive fragmentation of the mitochondrial network, suggesting a disruption in the mitochondrial dynamic balance. And to investigate the impact of the stress-induced disruption of mitochondrial fusion and fission dynamics on mitochondrial function, we assessed key functional markers, including Tomm20, cytochrome c (Cyto-c), and ROS. And a two-way ANOVA of Tomm20, Cyto-c, and ROS revealed the significant effects of ISO and Mdivi-1 treatment, ISO treatment, and Mdivi-1 treatment alone [Tomm20: (*F*(1, 24) = 11.68, *p* < 0.01), (*F*(1, 24) = 236.27, *p* < 0.01), and (*F*(1, 24) = 13.68, *p* < 0.01); Cyto-c: (*F*(1, 24) = 26.82, *p* < 0.01), (*F*(1, 24) = 319.51, *p* < 0.01), and (*F*(1, 24) = 23.10, *p* < 0.01); and ROS: (*F*(1, 24) = 18.91, *p* < 0.01), (*F*(1, 24) = 161.34, *p* < 0.01), and (*F*(1, 24) = 22.51, *p* < 0.01)]. After ISO treatment, post hoc tests showed that decreased Tomm20 (*p* < 0.01) and increased Cyto-c (*p* < 0.01) and ROS (*p* < 0.01). In contrast, ISO and Mdivi-1 treatment significantly increased Tomm20 (*p* < 0.01) (Figure 4a,b) and decreased Cyto-c (*p* < 0.01) (Figure 4d,e) and ROS (*p* < 0.01) (Figure 4f,g). Taken together, these findings demonstrated that stress impaired the mitochondrial function by disrupting the balance between fusion and fission, whereas the inhibition of excessive fission effectively restored mitochondrial functional integrity.

### 2.7. Expression of ER Stress Pathway Markers (IRE1α, ASK1, and JNK) in H9c2 Cardiomyocytes

**Figure 5:**
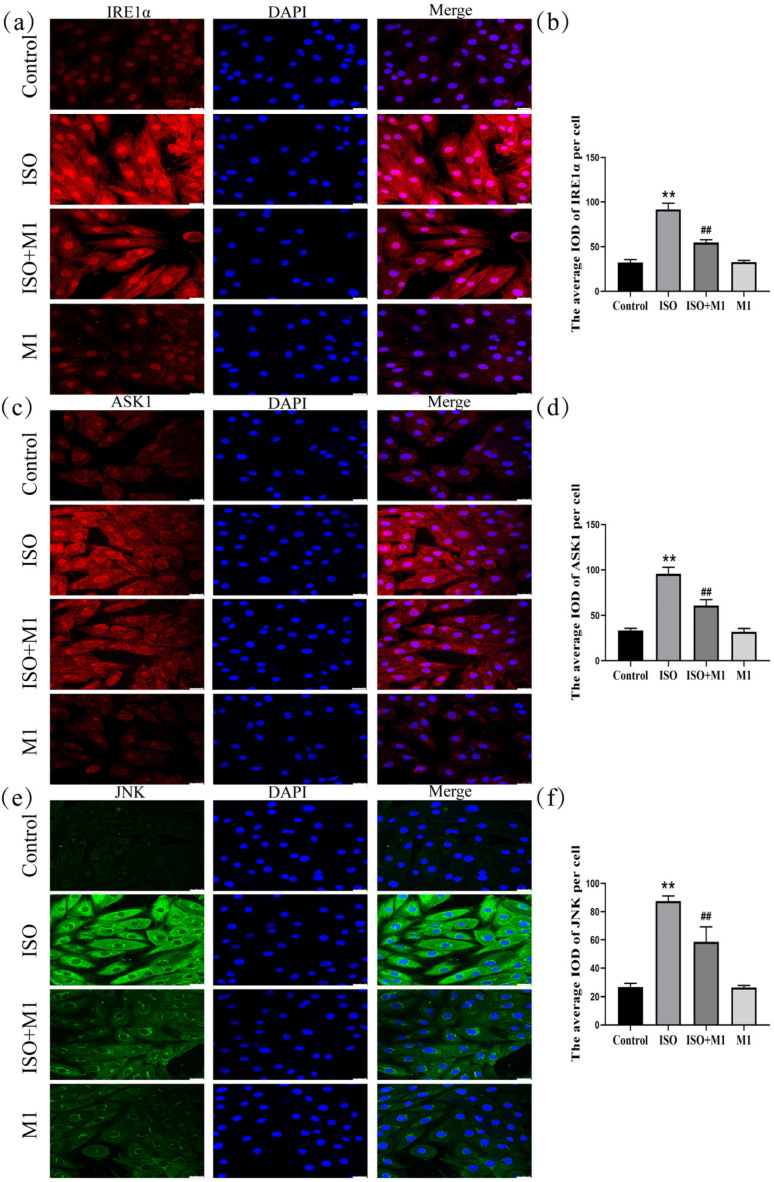
Expression of ER stress pathway markers (IRE1α, ASK1, and JNK) in H9c2 cardiomyocytes. (**a**,**b**) The expression of IRE1α in H9c2 cells. Scale bars = 25 μm. (**c**,**d**) The expression of ASK1 in H9c2 cells. Scale bars = 25 μm. (**e**,**f**) The expression of JNK in H9c2 cells. Scale bars = 25 μm. IOD = Integrated Optical Density. Data are presented as the mean ± SD (n = 6 independent biological experiments). ** *p* < 0.01 control vs. ISO group; ## *p* < 0.01 ISO vs. ISO + MI(Mdivi-1) group (two-way analysis of variance (ANOVA) with Tukey’s post hoc test).

Mitochondria dynamically interact and communicate with other organelles, most notably the endoplasmic reticulum. To investigate whether this interorganellar crosstalk participates in the stress response observed in our model, we examined the expression of key ER stress-associated proteins. A two-way ANOVA of ER stress proteins revealed significant effects on the interaction among ISO combined with Mdivi-1 treatment [IRE1α: (*F*(1, 24) = 127.03, *p* < 0.01); ASK1: (*F*(1, 24) = 57.88, *p* < 0.01); and JNK: (*F*(1, 24) = 35.49, *p* < 0.01)] in H9c2 cells, ISO treatment [IRE1α: (*F*(1, 24) = 591.46, *p* < 0.01); ASK1: (*F*(1, 24) = 428.64, *p* < 0.01); and JNK: (*F*(1, 24) = 383.89, *p* < 0.01)] in H9c2 cells, and Mdivi-1 treatment [IRE1α: (*F*(1, 24) = 123.12, *p* < 0.01); ASK1: (*F*(1, 24) = 69.59, *p* < 0.01); and JNK: (*F*(1, 24) = 37.56, *p* < 0.01)]. After ISO treatment, post hoc tests showed increased IRE1α (*p* < 0.01), ASK1 (*p* < 0.01), and JNK (*p* < 0.01). In contrast, ISO and Mdivi-1 treatment significantly decreased IRE1α (*p* < 0.01), ASK1 (*p* < 0.01), and JNK (*p* < 0.01) (Figure 5a–f). Collectively, these data suggested that, in our ISO-induced stress model, mitochondrial dynamic disturbance was associated with ER stress activation and that treatment with the fission inhibitor Mdivi-1 alleviated this response.

## 3. Discussion

The H9c2 rat cardiomyocyte cell line, cultured in a medium supplemented with 10% fetal bovine serum to promote a mature phenotype, was used as the model in the present study. ISO, a synthetic catecholamine analog, was applied to induce stress-related cardiomyocyte injury. CCK-8 assay results demonstrated a significant decrease in cell viability following ISO treatment. Moreover, desmin protein expression was downregulated in ISO-exposed cells. Given that desmin loss impairs myofibrillar stability and intercellular communication—key factors in pathological processes [23,24]—this observation further supports the validity of the injury model. Collectively, these data confirm that the established model is suitable for further mechanistic exploration.

In cardiomyocytes, the high abundance of mitochondria is dedicated to meeting substantial ATP requirements. Beyond this primary role, mitochondria are vital sensory organelles that detect internal and external cues, thereby mediating adaptive and survival responses through integrated energy and signaling networks [25,26]. The architecture of mitochondria is dynamically regulated by the opposing processes of fusion and fission, each mediated by specific proteins and associated with distinct functional outcomes [27,28]. Specifically, Mfn1 and Mfn2 mediate outer membrane fusion, while Opa1 drives inner membrane fusion [29,30,31]. Exposure to ISO induced a pronounced imbalance in mitochondrial dynamics within H9c2 cardiomyocytes. This dysregulation was characterized at the molecular level by the upregulation of pro-fission proteins (Drp1^ser616^, total Drp1, and Fis1) and downregulation of pro-fusion proteins (Mfn1, Mfn2, and Opa1). Such a molecular imbalance directly resulted in the structural disintegration of the mitochondrial network. Quantitative skeleton analysis based on Tomm20 immunofluorescence confirmed a significant increase in the mitochondrial fragmentation index under ISO stress compared to the normal control, thereby providing direct morphological evidence of disrupted mitochondrial dynamics. This dysregulation was further evidenced by a decrease in Tomm20, a key outer membrane receptor involved in protein import and metabolic competence [32,33], accompanied by elevated ROS and cytochrome c release. These collective results demonstrate that ISO-induced stress elicits mitochondrial dysfunction through the perturbation of mitochondrial dynamics.

Mitochondria form specialized contact sites with the endoplasmic reticulum (ER), known as mitochondria-associated ER membranes (MAMs), which play a critical role in regulating cellular activities in response to metabolic demands [34,35,36]. The disruption of mitochondrial function could impair this interorganellar signaling, thereby inducing ER stress—a condition characterized by an overload of unfolded or misfolded proteins within the ER lumen that exceeds its folding and degradative capacity [37,38]. During sustained ER stress, persistent activation of IRE1α initiates a downstream signaling cascade that culminates in JNK activation, a key mediator of cellular damage that is directly implicated in cell death [39,40,41]. In our ISO-induced H9c2 cardiomyocyte injury model, we observed the marked upregulation of IRE1α, ASK1, and JNK proteins concomitant with perturbations in mitochondrial dynamics, thereby establishing a causal link in which the ISO-mediated disruption of mitochondrial dynamics compromises mitochondria–ER communication, activates the IRE1α-ASK1-JNK pathway, and contributes to cardiomyocyte injury.

To further examine the interplay between disturbed mitochondrial dynamics and the IRE1α-ASK1-JNK ER stress pathway in vitro, we employed the mitochondrial fission inhibitor Mdivi-1 [42,43,44]. Mdivi-1 treatment significantly alleviated the imbalance in mitochondrial dynamics and associated injury, while also downregulating the expression of key components of the IRE1α-ASK1-JNK pathway. Previous in vitro and in vivo studies have demonstrated that, under hypoxic conditions, Mdivi-1 preserves mitochondrial structural integrity and inhibits ROS-driven ER stress activation, whereas ER stress inhibitors, such as PBA and TUDCA, despite having minimal effects on mitochondrial morphology, improve the function of pulmonary artery smooth muscle cells (PASMCs) under hypoxia [45,46,47]. Our findings align with these reports and further support the protective role of Mdivi-1 in ISO-simulated stress-induced myocardial injury, likely mediated through the stabilization of mitochondrial dynamics and attenuation of ER stress.

We acknowledge certain limitations of this study. First, our findings are derived primarily from the H9c2 cell line—a model that, despite its experimental convenience, does not fully recapitulate the metabolic and electrophysiological complexity of adult human cardiomyocytes. Second, the proposed mechanism—whereby ISO disrupts mitochondrial dynamics to drive ER stress—requires confirmation through genetic approaches. Employing techniques such as siRNA or CRISPR-Cas9 to modulate the expression of proteins involved in mitochondrial fission or the unfolded protein response (UPR) in future studies will be essential to substantiate the causal pathway identified here.

In summary, our study found that high concentrations of ISO reduced H9c2 cardiomyocyte viability and induced cardiomyocyte injury. In the ISO-established model of stress-induced myocardial injury, ISO triggered the disruption of mitochondrial fusion/fission dynamics, which promoted endoplasmic reticulum stress and activated the IRE1α-ASK1-JNK signaling pathway, thereby contributing to cardiomyocyte damage. These findings provide a pathological and morphological basis for understanding cardiomyocyte injury induced by catecholamine-like substances. Furthermore, by focusing on mitochondrial morphological alterations, this work offers novel mechanistic perspectives for understanding the pathological processes underlying stress-related cardiovascular diseases and for developing potential preventive and therapeutic strategies.

## 4. Materials and Methods

### 4.1. Materials

The rat H9c2 cardiomyocyte cell line was procured from the National Collection of Authenticated Cell Cultures (Shanghai, China; Catalog No. GNR 5). Cells were cultured in high-glucose Dulbecco’s Modified Eagle Medium (DMEM) sourced from Gibco (Grand Island, NY, USA), which was supplemented with 10% fetal bovine serum (FBS; Corning, NY, USA) and 1% penicillin/streptomycin (100 U/mL; Sigma-Aldrich, St. Louis, MO, USA). Cell freezing medium was obtained from Biological Industries (Beit HaEmek, Israel). For experimental assessments, cell viability and intracellular reactive oxygen species (ROS) levels were determined using the Cell Counting Kit-8 (CCK-8) and ROS Assay Kit, respectively (both from Beyotime Biotechnology, Shanghai, China). The following primary antibodies were employed: desmin (Proteintech, San Diego, CA, USA, CL594-16520), Drp1 (Abcam, Cambridge, UK, ab184247), phospho-Drp1 (Ser616) (Affinity, Shanghai, China, AF8470), Fis1 (ABclonal, Woburn, MA, USA, A19666), Mfn1 (Proteintech, 13798-1-AP), Mfn2 (Proteintech, 12186-1-AP), Opa1 (Proteintech, 27733-1-AP), Cyto-c (Proteintech, 10993-1-AP), Tomm20 (Abcam, ab186735), IRE1α (Proteintech, 27528-1-AP), ASK1 (Proteintech, 28201-1-AP), and JNK (Proteintech, 66210-1-lg). The mitochondrial fission inhibitor Mdivi-1 was acquired from MedChemExpress (Monmouth Junction, NJ, USA, HY-15886), and isoproterenol (ISO) was sourced from Sigma-Aldrich. Fluorescently labeled secondary antibodies (Donkey anti-Mouse and Donkey anti-Rabbit IgG (H + L)), conjugated to either Alexa Fluor™ 488 or 594, were procured from Thermo Fisher Scientific (Carlsbad, CA, USA).

### 4.2. Establishment of Stress-Induced H9c2 Cardiomyocyte Injury Model

H9c2 rat cardiomyocytes were cultured under standard conditions (37 °C, 5% CO_2_) in high-glucose DMEM (Gibco, USA) containing 10% FBS (Corning, Corning, NY, USA) and 100 U/mL penicillin/streptomycin (Sigma, USA). A stress-induced injury model was generated by subjecting the cells to 50 μM isoproterenol (ISO) for 24 h. The role of mitochondrial dynamics in this process was probed through the pharmacological inhibition of fission using Mdivi-1. H9c2 cells were pretreated with 50 µM Mdivi-1 for 2 h prior to a 24 h co-incubation with ISO. Accordingly, the experimental design consisted of four groups: control, ISO, ISO + Mdivi-1, and Mdivi-1-only group.

### 4.3. Cell Viability Detection

Quantification of viability in differentiated H9c2 cardiomyocytes was conducted utilizing the CCK-8 assay. After plating in 96-well plates (5 × 10^3^ cells/well), the cell cultures were subjected to isoproterenol (ISO) treatment at specified concentrations (12.5, 25, 50, 150, and 200 μM) for durations of 24, 48, and 72 h. The assay involved the supplementation of each well with 10 μL of CCK-8 solution and subsequent incubation for 4 h at 37 °C. Absorbance measurements at 450 nm were obtained with a Multiskan GO microplate reader (Thermo Fisher Scientific, Waltham, MA, USA). The final viability values were computed based on the following equation: [OD (experimental) − OD (blank)]/[OD (control) − OD (blank)].

### 4.4. ROS Detection

The generation of reactive oxygen species (ROS) was assessed using the ROS Assay Kit (Beyotime, Shanghai, China). H9c2 cells were cultured in 6-well plates and treated with 10 μmol/L DCFH-DA in serum-free medium at 37 °C for 20 min. Following treatment, the plates were washed three times with serum-free medium. Intracellular ROS fluorescence was captured using a fluorescence microscope and analyzed with Image software (version 1.54).

### 4.5. Immunofluorescence Staining

H9c2 cardiomyocytes destined for confocal imaging were seeded in 24-well glass-bottom plates at 1 × 10^5^ cells per well. Subjected to ISO treatment, the cells underwent a standardized immunofluorescence procedure: two washes with PBS, fixation in ice-cold 4% paraformaldehyde (20 min), and subsequent permeabilization with 0.1% Triton X-100 in 2% BSA (15 min). Non-specific sites were blocked with 2% BSA (1 h, room temperature) prior to an overnight incubation at 4 °C with appropriately diluted primary antibodies. Corresponding secondary antibodies (Donkey anti-Mouse Alexa Fluor™ 488 and Donkey anti-Rabbit Alexa Fluor™ 594; Thermo Fisher Scientific) were applied thereafter. Visualization was carried out on a Zeiss LSM700 confocal microscope (Zeiss, Jena, Germany). Quantification was performed on at least 40 cells per field from 5 randomly selected fields (scale bar = 25 μm).

### 4.6. Analysis of Mitochondrial Fragmentation Index

Mitochondrial morphology was quantitatively analyzed based on Tomm20 immunofluorescence images. Acquired images were processed using Image J software for binarization and skeletonization, and the “mitochondrial network fragmentation index” was calculated. This index is defined as the ratio of the total number of skeletonized branches to the total mitochondrial network area (Fragmentation Index = Total Number of Branches/Total Network Area), serving as an objective metric for assessing network fragmentation. Images from four independent biological replicates were analyzed for each experimental group.

### 4.7. Statistical Analysis

All experiments were performed with biologically independent samples and repeated at least three times (with each experimental group containing independent replicates per repeat). Data are presented as mean ± standard deviation (SD). Normality was assessed using the Shapiro–Wilk test, and homogeneity of variances was verified with Levene’s test. For datasets meeting these assumptions, statistical comparisons between groups were performed using one-way or two-way analysis of variance (ANOVA), followed by Tukey’s honestly significant difference (HSD) post hoc test for multiple comparisons. All analyses were conducted in SPSS 21.0 (IBM SPSS Statistics, Chicago, IL, USA). *p* < 0.05 was considered statistically significant.

## Data Availability

The original contributions presented in this study are included in the article. Further inquiries can be directed to the corresponding authors.
